# Ginger (*Zingiber officinale*) Root Capsules Enhance Analgesic and Antioxidant Efficacy of Diclofenac Sodium in Experimental Acute Inflammation

**DOI:** 10.3390/antiox12030745

**Published:** 2023-03-18

**Authors:** Ioana Boarescu, Raluca Maria Pop, Paul-Mihai Boarescu, Ioana Corina Bocșan, Dan Gheban, Adriana Elena Bulboacă, Anca Dana Buzoianu, Sorana D. Bolboacă

**Affiliations:** 1 Department of Medical Informatics and Biostatistics, Iuliu Haţieganu University of Medicine and Pharmacy Cluj-Napoca, Louis Pasteur Street, No. 6, 400349 Cluj-Napoca, Romania; 2 Department of Pharmacology, Toxicology and Clinical Pharmacology, Iuliu Haţieganu University of Medicine and Pharmacy Cluj-Napoca, Gheorghe Marinescu Street, No. 23, 400337 Cluj-Napoca, Romania; 3 Department of Pathological Anatomy, Iuliu Haţieganu University of Medicine and Pharmacy Cluj-Napoca, Clinicilor Street, No. 3-5, 400006 Cluj-Napoca, Romania; 4 Department of Pathophysiology, Iuliu Haţieganu University of Medicine and Pharmacy Cluj-Napoca, Victor Babeş Street, No. 2-4, 400012 Cluj-Napoca, Romania

**Keywords:** ginger, capsules, analgesic, antioxidant

## Abstract

Our study aimed to evaluate the analgesic and antioxidant effects of ginger (*Zingiber officinale*) root capsule extract (GRCE) in addition to diclofenac (D) sodium treatment in carrageenan-induced acute inflammation (AI). Seven groups of eight Wistar-Bratislava white rats were included in the study. One group was the control (C), and AI was induced in the other six groups. The following treatments were applied: saline solution for C and AI groups, D for the AID group, GRCE for two groups and GRCE and D for another two groups. The GRCE was administered by gavage in two doses (100 mg/Kg b.w. or 200 mg/kg b.w.), while D was administered intraperitoneally in a dose of 5 mg/kg b.w. The association of GRCE with this low dose of diclofenac reduced pain threshold and improved mobility with the best results for the dose of 200 mg/kg b.w. Moreover, this combination reduced, better than D alone, the serum levels of the evaluated pro-oxidant parameters (malondialdehyde, the indirect assessment of NO synthesis, total oxidative status and oxidative stress index) up to 78%, especially oxidative stress index (*p* < 0.0001). GRCE alone slightly improved the antioxidant parameters (total antioxidant capacity and total thiols), but when associated with, D the results were better, especially for total thiols as their plasma levels increased up to 50% (*p* < 0.0010), with the best results obtained for the 200 mg/kg b.w. dose of GRCE. In conclusion, ginger root capsules associated with diclofenac might offer additional antioxidant and analgesic effects in a dose-dependent manner in acute inflammation.

## 1. Introduction

The carrageenan-induced paw edema is the most commonly used animal model of acute inflammation [1]. Moreover, it is a well-defined model used to evaluate the anti-inflammatory and anti-edematous potential of pharmacological substances, as there is a variety of inflammatory mediators involved in its development [2,3,4,5].

Acute inflammation has two main components: vascular changes associated with cellular events. Carrageenan is a natural linear sulfated polysaccharide and the sulfated sugars present in carrageenan are responsible for the production of vascular and cellular events of inflammation due to the activation of inflammatory mediators [6]. Oxidative has a major impact in the pathophysiological mechanisms of acute inflammation, as it can activate various transcription factors, leading to differential expression of some genes involved in the inflammatory pathways [4]. Oxidative stress is defined as an imbalance between the production of reactive oxygen species (ROS) and their neutralization by the antioxidant system. Moreover, this imbalance can be responsible for damaging cellular molecules such as deoxyribonucleic acid (DNA), lipids or proteins [7].

Medications such as nonsteroidal anti-inflammatory drugs (NSAIDs) are commonly used in the management of acute inflammation. Diclofenac (2-[(2,6-dichlorophenyl)amino] benzenacetic acid), a well-known NSAID drug, exerts its anti-inflammatory effects through the inhibition of the arachidonate metabolites synthesis secondary to cyclooxygenase (COX) inhibition [8]. Diclofenac was observed to possess dose–response relationships for COX-2 and COX-1 inhibition, with greater COX-2 selectivity [9]. Moreover, it was observed to significantly reduce the production of pro-inflammatory cytokines, such as Tumor Necrosis Factor-α (TNF-α) and Interleukin-6 (IL-6) in acute inflammation [10]. Administration of NSAIDs may cause side effects such as hypertension, acute renal failure, gastrointestinal ulcers, serious cardiovascular events and even worsen preexisting heart failure. Limitation of NSAIDs’ side effects can be achieved by a reduction in dosage and treatment duration [11].

Medicinal plants have been used throughout history as a popular method of therapy for pain relief [12]. *Zingiber officinale* roscoe (Z. *officinale*), commonly known as ginger, is a member of the *Zingiberaceae* family and has been widely used as a spice [13,14]. Major biologically active compounds, such as gingerols, shogaols and paradols can be found in *Zingiber officinale*, but the chemical analysis shows that it contains more than 400 different compounds [15]. In experimental studies conducted in rodents, *Zingiber officinale* was reported to have various therapeutic effects such as anti-emetic in cancer chemotherapy, hypoglycemic in and streptozotocin-induced diabetes mellitus model and anti-inflammatory in egg albumin-induced pedal edema [16,17,18]. Moreover, it was observed to attenuate muscle pain significantly [19] and to reduce injury-induced neuropathic pain [20,21] and oxaliplatin-induced neuropathic pain [22].

Various in vivo and in vitro tests have explored the anti-oxidative properties of ginger and its components [23,24,25,26]. In an animal model study, it was shown that ginger significantly raised the levels of antioxidant enzymes, together with serum glutathione and lowered induced lipid peroxidation [24]. Among its components, 6-Shogaol was observed to exhibit the most potent antioxidant and anti-inflammatory properties in ginger, these effects being attributed to the presence of the alpha, beta-unsaturated ketone moiety [25]. Another component, 6-gingerol, might have an enhanced antioxidant effect in protection from oxidative damage caused by free ROS, as a result of its free radical-scavenging ability [26].

Gingerol, shogaol and other structurally-related compounds in ginger express their anti-inflammatory effects through inhibition of the prostaglandin and leukotriene biosynthesis, as a result of 5-lipoxygenase or prostaglandin synthetase suppression [23]. The inhibition of the pro-inflammatory cytokines such as Interleukin-1 (IL-1), TNF-α and Interleukin-8 (IL-8) was described as another anti-inflammatory mechanism observed for ginger [27,28]. Moreover, it was already reported that shogaol can down-regulate inflammatory inducible nitric oxide synthase (iNOS) and cyclooxygenase-2 (COX-2) gene expression in macrophages [29].

Our study aimed to evaluate the analgesic and antioxidant effects of ginger (*Zingiber officinale*) root capsule extract in addition to diclofenac sodium in carrageenan-induced acute inflammation.

## 2. Materials and Methods

The study was conducted in accordance with the Declaration of Helsinki and approved by the Ethics Committee of the Iuliu Hațieganu University of Medicine and Pharmacy Cluj-Napoca (approval no. 25/3 February 2021) and by the Sanitary-Veterinary and Food Safety Directorate from Cluj-Napoca (approval no. 252/17 March 2021).

### 2.1. Chemicals and Drugs

Saline solution (0.9%) and diclofenac sodium injection were purchased from a local pharmacy in Cluj-Napoca. 

### 2.2. Plant Material

Ginger root capsule extract (GRCE) (Solaray, Park City, UT, USA) was purchased from a local pharmacy where the capsules were commercialized as a food supplement. As stated in the pamphlet, the 250 mg capsules have the following ingredients: ginger (Zingiber officinale) (root extract) (guaranteed 12.5 mg (5%) gingerols), ginger (Zingiber officinale) (root) 100 mg, magnesium carbonate, vegetable cellulose capsule, maltodextrin, magnesium stearate, silica and croscarmellose sodium.

### 2.3. Extraction of Ginger Root Capsules

The content of 10 ginger root capsules was extracted with 20 mL ethanol on a magnetic stirrer for 1 h. Afterward, the mixture was kept in dark conditions at 4 °C for 24 h, followed by filtration (Whatman filter paper no.3). The pellet was resuspended again in 10 mL ethanol and the mixture was sonicated for 30 min at room temperature and filtered (Whatman filter paper no.3). The ginger root capsule extract (GRCE) was further analyzed for its phytochemicals compounds, total polyphenols content and total antioxidant capacity. 

### 2.4. Total Polyphenol Content 

Total polyphenols content (TPC) was evaluated using Folin–Ciocalteu as previously described by Pop et al. [30]. Ginger root capsules extract (25 μL of) was mixed with Folin–Ciocalteu reagent (125 μL; 0.2 N) and sodium carbonate (Na_2_CO_3_) solution (100 μL; 7.5% *w*/*v*), homogenized, put in 96-well plates and incubated at room temperature in dark conditions. After 2 h of incubation, the plates were read at 760 nm using the Microplate Reader Synergy HT Multi-Detection (BioTek Instruments, Inc., Winooski, VT, USA). The results were expressed as gallic acid equivalents (GAE) using a gallic acid calibration curve (r^2^ = 0.9946). The analysis of GRCE was performed in triplicate and expressed as mean values (mg/g dry weight (d.w.) of extracts) ± standard deviations.

### 2.5. Radical-Scavenging Capacity Antioxidant Capacity Test

The radical-scavenging capacity (DPPH) of GRCE was performed following the Brand-Williams method [31]. Accordingly, 250 μL of GRCE sample was mixed with 1750 μL of 0.02 mg/mL DPPH solution and incubated at room temperature (30 min). The absorbance was recorded by a 96-well plates Synergy HT Multi-Detection Microplate Reader (BioTek Instruments, Inc., Winooski, VT, USA) at 517 nm. The control was performed with methanol. A standard calibration curve with Trolox (r^2^ = 0.9985) was used for the interpretation of the results, which were expressed as Trolox equivalents (TE) per 100 g dry weight (d.w.). The experiments were performed in triplicates. 

### 2.6. High-Performance Liquid Chromatography-Diode Array Detection–Electro-Spray Ionization Mass Spectrometry Analysis of Ginger Root Capsule Extract

The High-Performance Liquid Chromatography–Mass Spectrometry (HPLC-MS) analysis of GRCE was performed as described by Pop et al. [30]. Agilent 1200 HPLC with DAD detection was coupled to Agilent 6110 single quadrupole mass spectrometer. The column used was Eclipse XDB C18 (4.6 × 150 mm, 5 m particle size) from Agilent Technologies, Santa Clara, CA, USA. The separation was performed at room temperature using a gradient by mixing mobile phase A (0.1% acetic acid in distilled water (99:1) (*v*/*v*)) and mobile phase B (0.1% acetic acid in acetonitrile (*v*/*v*)) [32]. The elution gradient is presented in Table 1.

The spectra were registered at 280 nm and further injected into the MS equipped with an ESI source and scanned between 100 and 1000 *m*/*z*. The compound’s ionization was performed in the (+) mode at 350 °C. The nitrogen flow was set at 8 L/min and the capillary voltage at 3000 V. Agilent Chem-Station Software (Rev B.04.02 SP1, Palo Alto, CA, USA) was used for data analysis. The tentative compound identification was performed considering mass spectra, UV−visible spectra, retention time and the literature data.

### 2.7. Animals 

Sixty-two (62), ten-week-old, white male Wistar-Bratislava rats (300–320 g) were included in the study. They were all purchased from the Animal Department of the Faculty of Medicine, Iuliu Haţieganu University of Medicine and Pharmacy. They were acclimatized to standard environmental conditions of 22–25 °C, 30% humidity and 12 h/12 h light/dark cycle, having free access to water and food.

### 2.8. Toxicity Testing

Six rats were used to evaluate the toxicity of ginger root capsules (GRCE) according to the recommendations of the guideline for testing chemicals issued by the Organization for Economic Co-operation and Development (OECD) [33]. 

The content of GRCE was dissolved in saline solution and administrated orally by gavage. Initially, a dose of 50 mg/kg b.w. (body weight) was administered to 3 rats. Each rat was observed individually after administration of the ginger solution at least once in the first 30 min and periodically in the first 24 h, with special attention given in the first 4 h. Afterwards, all rats were observed daily for 14 days. Possible changes in the skin and fur, eyes and mucous membranes, as well as the cardiac, respiratory and nervous system, as well as behavioral disorders, were monitored. Close attention was paid in order to observe whether the rats exhibited tremors, convulsions, excessive salivation, diarrhea, lethargy or drowsiness. The weight of the rats was determined before the administration of the extract and thereafter once every 7 days up to 14 days. At the end of the experiment (day 14), under local anesthesia with xylazine and ketamine, blood samples were collected from each rat and toxicity tests consisted of evaluating the serum levels of alanine aminotransferase (ALT), total bilirubin (TB), creatinine and urea. The rats were sacrificed and their liver and kidneys were taken, fixed in 10% formalin. After fixation in paraffin, stained with hematoxylin and eosin, a pathologist examined the tissue fragments under a light microscope. Since no rat died, another 3 rats and the same steps were followed to test the 300 mg/kg b.w. GRCE dose for another 14 days, and afterward another 3 rats for the 2000 mg/kg b.w. dose for another 14 days. 

### 2.9. Experimental Design

Since there were no significant differences between their weights, the fifty-six (56) rats were randomly divided into eight groups of seven animals each and treated as follows:

(1) C, the control group, rats had no intervention and were treated with saline solution;
(2) Acute inflammation (AI) group, acute paw inflammation was induced and rats were treated with saline solution;
(3) AI treated with diclofenac sodium (AI-D) group, acute paw inflammation was induced and rats were treated with diclofenac sodium (5 mg/kg b.w.);
(4) AI treated with GRCE in the lower dose (AI-GRCE100) group, acute paw inflammation was induced and rats were treated with GRCE in a dose of 100 mg/kg b.w.;
(5) AI treated with GRCE in the higher dose (AI-GRCE200) group, acute paw inflammation was induced and rats were treated with GRCE in a dose of 200 mg/kg b.w.;
(6) AI treated with GRCE in the lower dose and D (AI-GRCE100-D) group, acute paw inflammation was induced and rats were treated with GRCE in a dose of 100 mg/kg b.w., and D in a dose of 5 mg/kg b.w.;
(7) AI treated with GRCE in the higher dose and D (AI-GRCE200-D) group, acute paw inflammation was induced and rats were treated with GRCE in a dose of 200 mg/kg b.w. and D in a dose of 5 mg/kg b.w.

Acute inflammation was induced using 100 μL of 1% freshly prepared carrageenan solution, on day 0 of the experiment. Carrageenan solution was injected sub-plantary into the right-hind paw [34].

Only one dose of diclofenac sodium of 5 mg/kg b.w. was administered intraperitoneal (i.p.) right after AI induction. C and AI groups received 1 mL of saline solution i.p. The reduced dose of 5 mg/kg b.w. of diclofenac sodium was used as it was previously observed to reduce paw edema in carrageenan-induced AI [35,36]. The GRCE was dissolved in saline solution and administrated orally by gavage right after diclofenac administration. Control and AI groups received 1 mL of saline solution by gavage. A dose of 100 mg/kg b.w. was chosen as this dose of *Zingiber officinale* was proven to have antioxidant and anti-inflammatory effects [37] and the dose of 200 mg/kg b.w. was proven to have analgesic effects [38]. 

### 2.10. Outcome Measurements 

The physical tests described in Table 2 (paw pressure, hot plate and motility tests) were performed at 1, 3, 5, 7 and 24 h after carrageenan administration. 

The animal care staff, those who administered the treatment and those who collated data during motility, paw pressure and hot plate tests were unaware of allocation groups. Neither the persons involved in blood sample collection, biochemical and histological analysis, nor the person who did the statistical analysis were aware of the treatment received by each rat.

### 2.11. Blood Samples and Biochemical Assays

Under light anesthesia with xylazine and ketamine, the blood samples were collected from the retro-orbital plexuses of each rat, at 24 h after AI induction, in heparinized tubes (Startstedt AG and Co., Nümbrecht, Germany). Plasma was obtained by centrifugation at 4 °C for 20 min at 16,200× *g*, transferred in Eppendorf tubes and kept at −80 °C until further analysis. 

The serum levels of ALT, TB, urea and creatinine were determined using an automatic analyzer Applied Biosystem (Costa Brava, Barcelona, Spain) through a spectrophotometric method.

Five oxidative stress parameters were assessed from plasma with a Jasco V-530 UV–Vis spectrophotometer (Jasco International Co. Ltd., Tokyo, Japan), using the methods previously described: malondialdehyde (MDA) [42], the indirect assessment of NO synthesis (NOx) [43], total oxidative status (TOS) [44], total antioxidant capacity (TAC) [45], total thiols (SH) [46] and oxidative stress index (OSI) [47]. 

### 2.12. Statistical Methods

Means and standard deviations were used as descriptive statistics in reporting the primary outcomes, namely the serum levels of the evaluated markers (MDA, NOx, TOS, TAC, total thiols and OSI). The same descriptive statistics indicators were used for secondary outcomes, namely paw pressure and hot plate tests while for the motility test, we reported percentages associated with the scores. Student t-test for independent groups was used to test the induction of AI comparing the C group with the AI group as well as the effects of the low D dose on evaluated markers and signs comparing the AI group with the AIC group. The anti-inflammatory and antioxidant effects of the GRCE with or without D were compared with an ANOVA test followed by post hoc analysis using the Scheffe test (at a significance level of 0.008) whenever data proved statistical differences on a two-tailed test at a significance level of 5%. The distribution of raw data of the evaluated serum markers was graphically represented using a variability plot that shows individual values along with the median. The results of the paw pressure and hot plate tests were graphically represented using the mean and 95% confidence interval for each group. The distribution of the motility test was represented with a 100% stacked bar per group.

Statistical analysis was conducted blinded so that the treatment group was not identifiable during the analysis. The correspondence between the code and the group’s name was conducted when the article was written. Data analysis was conducted with Statistica software (v. 13.5, StatSoft, St Tulsa, OK, USA).

## 3. Results

### 3.1. Ginger Root Capsules Extract Phytochemical Analysis

**Figure 1:**
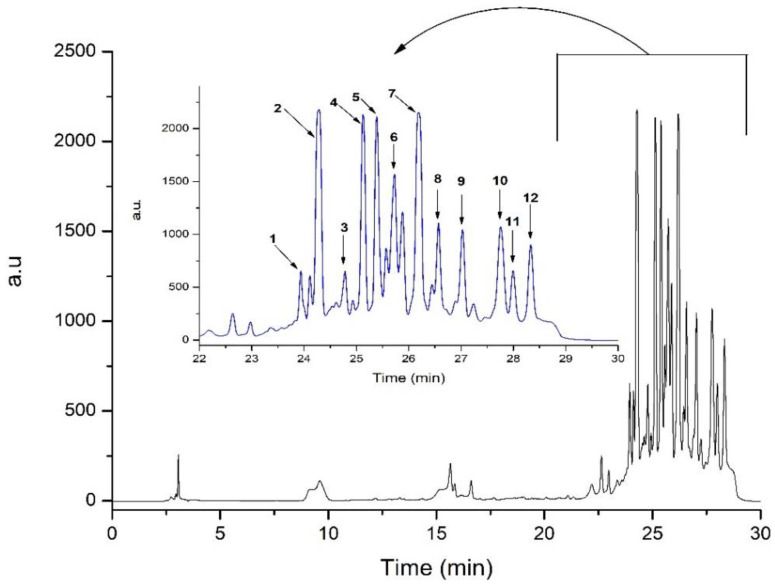
HPLC chromatogram of ginger root ethanol extract registered at 280 nm. The identification of the main compounds is listed in Table 3.

The GRCE total polyphenols content (TPC) was 3757.45 ± 58.57 mg GAE/100 g d.w. plant material, while the total antioxidant capacity was 0.918 ± 0.01 mM Trolox Equivalents/100 g d.w. The HPLC-DAD-ESI MS identified significant concentrations of gingerols and gingerol derivatives, gingerdiols, gingerdiones and shogaols, the principal classes of ginger compounds (Table 1 and Figure 1).

### 3.2. Ginger Root Capsules Extract Toxicity

No changes in the skin and fur, eyes or mucous membranes were observed in rats who received the dose of 50 mg/kg b.w., 300 mg/kg b.w. or 2000 mg/kg b.w. of GRCE. Neither heart rate, respiratory rate or neurological/behavioral disorders were observed in all 3 doses. A slight increase in weight was observed in all three groups after 2 weeks. No significant variations were observed in any liver or kidney evaluated serum markers, at the tested doses, as shown in Table 4.

**Figure 2:**
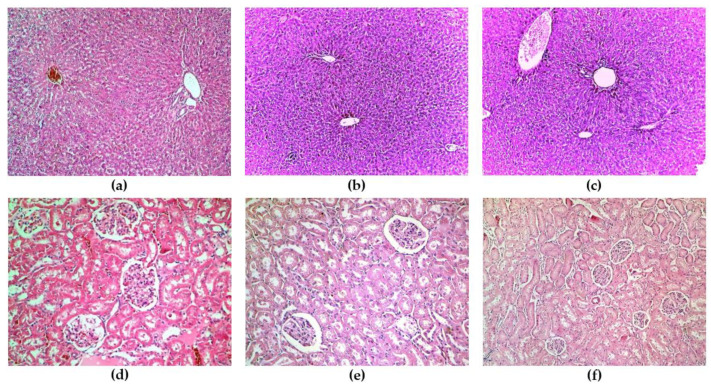
Histological examination: (**a**) liver—dose of 50 mg/kg b.w. (**b**) liver—dose of 300 mg/kg b.w., (**c**) liver—dose of 2000 mg/kg b.w., (**d**) kidney—dose of 50 mg/kg I., (**e**) kidney—dose of 300 mg/kg b.w. and (**f**) kidney—dose of 2000 mg/kg b.w.

Histological examination revealed the normal architecture of the liver and kidneys of each rat used for toxicity testing, as shown in Figure 2.

### 3.3. Effects of Acute Inflammation on the Evaluated Oxidative Stress Serum Markers and the Effects of Diclofenac on These Markers

The MDA, NOx and TOS values significantly increased and TAC and total thiols significantly decreased after AI induction (Table 5). Diclofenac significantly decreased (MDA, NOx and TOS) and, respectively, increased the TAC serum values showing antioxidant efficacy (Table 5). No significant changes in the AI-D group than in AI were observed on serum values of total thiols (Table 5). 

**Figure 3:**
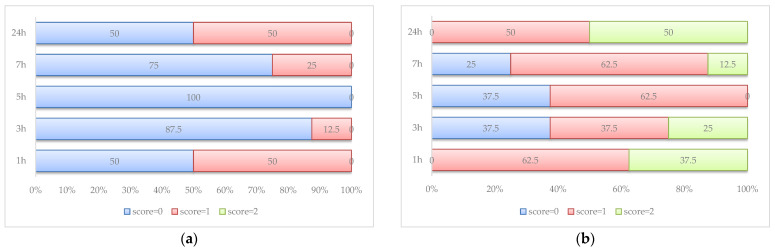
Motility scores (percentages) in AI group (**a**) and AI-D group (**b**) (score 0 = rat walked with difficulty and avoided touching the toes of the inflamed paw to the floor; score 1 = rat walked with little difficulty, but it touched the floor with the toe of the inflamed paw; score 2 = rat walked easily without any difficulties).

Without any exception, the rats walked easily without any difficulties on the motility test in the control group (score = 2). Diclofenac exhibits its effect on motility, with 50% of rats walking without any motility difficulties at 24h after AI induction (Figure 3).

### 3.4. Ginger Enhancement of the Diclofenac Analgesic and Antioxidant Efficacy

**Figure 4:**
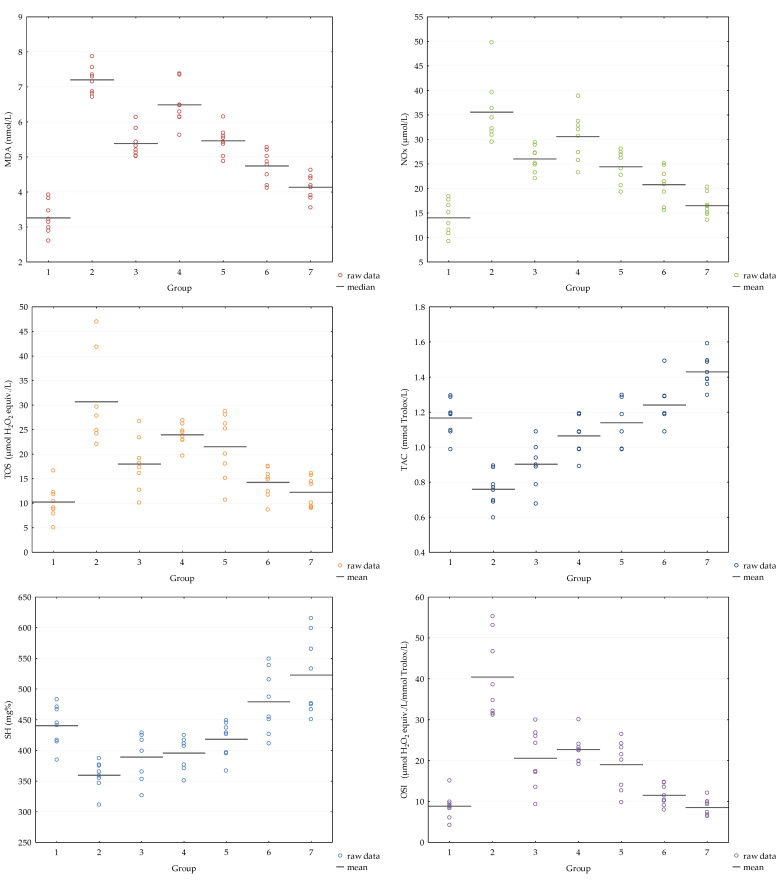
Variation of evaluated serum markers by group (1 = C, 2 = AI, 3 = AI-D, 4 = AI-GRCE100, 5 = AI-GRCE200, 6 = AI-GRCE100-D, 7 = AI-GRCE200-D).

The AI-GRCE200-D group obtained the closest serum values of the evaluated markers to the control group, with statistically significant differences between groups (Table 6, Figure 4).

**Figure 5:**
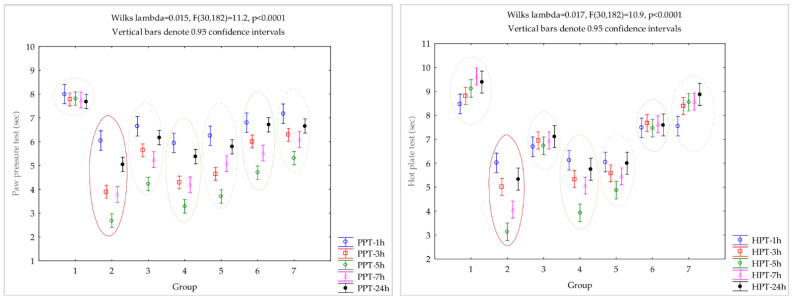
Variation of measurements obtained by paw pressure test (PPT, left graph) and hot plate test (HPT, right graph) by group (1 = C, 2 = AI, 3 = AI-D, 4 = AI-GRCE100, 5 = AI-GRCE200, 6 = AI-GRCE100-D, 7 = AI-GRCE200-D).

The better results on paw pressure tests were obtained by AI-GRCE100-D and AI-GRCE200-D groups, with the closest values to the control group and slightly better results compared to the AI-D group (Figure 4). The AI-GRCE200-D group shows better results on the hot plate test, with the closest values to the control group (Figure 5).

**Figure 6:**
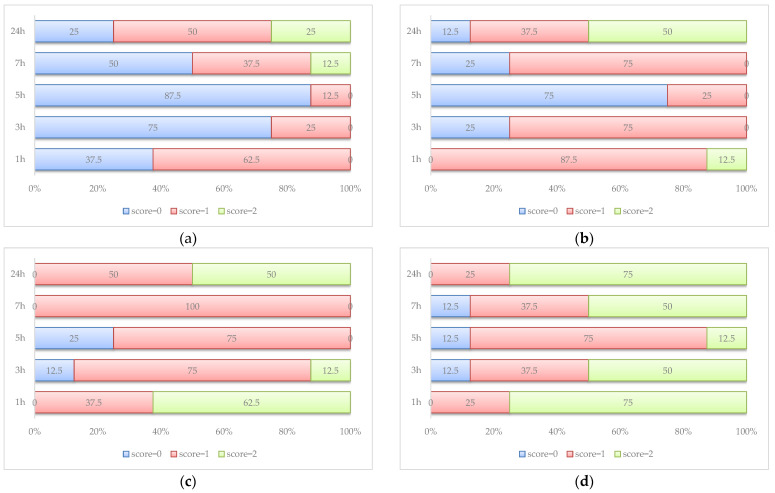
Percentage distribution of motility scores by group: (**a**) AI-GRCE100 group; (**b**) AI-GRCE200 group; (**c**) AI-GRCE100-D group; and (**d**) AI-GRCE200-D group. Score 0 = rat walked with difficulty and avoided touching the toes of the inflamed paw to the floor; score 1 = the rat walked with little difficulty, but it touched the floor with the toe of the inflamed paw; score 2 = rat walked easily without any difficulties.

The best motility is observed in the rats in the AI-GRCE200-D group (Figure 6). Normal motility at 24 h was observed on 6/8 rats in the AI-GRCE200-D group, while half of the rats in the AI-GRCE200 and AI-GRCE100-D groups showed normal motility at the same measurement.

## 4. Discussion

### 4.1. Ginger Root Capsules Extract Phytochemical Analysis and Toxicity

Phytochemical analysis of GRCE revealed significant concentrations of 6-gingerol, 8-gingerol, 10-gingerol and 6-shogaol (Table 3) similar to the capsules evaluated by Zick et al. in healthy human subjects [48].

No clinical signs of toxicity nor biochemical or histological abnormal results were observed for the three doses of the GRCE (50mg/kg b.w., 300 mg/kg b.w. or 2000 mg/kg b.w.). Toxicity testing of ginger (*Z. officinale*) powder capsule performed by Zick et al. [48] in healthy human subjects reported no adverse events for the dose of 100 mg. Higher doses of 1000 mg or 2000 mg were associated with minor gastrointestinal symptoms, including eructation, heartburn and indigestion, but no toxicities greater than the National Cancer Institute Common Toxicity Criteria (version 2.0) grade 1 were reported [48].

### 4.2. Ginger Root Capsule Extract Enhancement of the Diclofenac Analgesic and Antioxidant Efficacy

The results of the present study demonstrate that the association of ginger with diclofenac sodium provides dose-dependent analgesic and additional antioxidant effects in carrageenan-induced acute inflammation (Figure 4, Figure 5 and Figure 6, Table 6). To the best of our knowledge, this is the first study focused on evaluating the additional analgesic and antioxidant efficacy of ginger (*Zingiber Officinale*) root capsule extract to diclofenac sodium in experimental acute inflammation.

The administration of GRCE alone slightly improved the motility score but when it was associated with D, this combination better improved the motility score (Figure 6) more than D alone, most probably due to the fact that GRCE offers supplementary analgesic and anti-inflammatory effects. It was already observed that ginger injected intraperitoneally can effectively decrease disease incidence, joint temperature and swelling, and ameliorate clinical scores in rats with collagen-induced arthritis, with the best results for the dose of 200 mg/kg b.w. [49]. The anti-inflammatory effects of ginger are the result of the inhibition of the induction of several genes involved in the inflammatory response (e.g., genes encode the inducible cyclo-oxygenase-2 enzyme, chemokines and cytokines) [50]. 

The paw pressure test is a useful method for evaluating nociceptive thresholds, often used to test the effectiveness of different analgetics by observing the reaction to gradually increasing pressure on the inflamed paw [51]. Ginger administration was observed to provide a reduced analgesic effect (Table 6, Figure 5); moreover, it was already suggested that on mechanically induced pain the analgesic effects of ginger are dose-dependent [52]. In our study, the best analgesic effect was obtained after the association of GRCE in the dose of 200 mg/kg b.w. with D. Diclofenac administration was already demonstrated to increase the withdrawal threshold in paw pressure tests and, therefore, to provide analgesic effects [35]. Diclofenac, as a nonsteroidal anti-inflammatory drug (NSAID), reduces the inflammation process and therefore the associated pain [10]. Our results suggest that the 5 mg/kg b.w. dose of diclofenac sodium might have limited anti-nociceptive effects (Table 6, Figure 5) because it offers a reduced dose of the active substance. Ginger antinociceptive activity might be related to the inhibition of arachidonic acid synthesis, a metabolite that is mediated by COX inhibition [12].

The hot plate test is a thermoanalgesic method useful to evaluate the central activity of different analgesic drugs, since, in this test, the response reflex is mediated by supraspinal centers [53]. Our results show that GRCE administration was observed to provide a slight thermoanalgesic effect compared to D. Better results were observed after the combination of GRCE with D (Table 6, Figure 5). Diclofenac is an NSAID, so it has analgesic effects proved by the elevation of time to paw withdrawal to thermal stimuli, a behavior observed as well as in previous studies [54,55]. *Zingiber officinale* dried rhizomes ethanol extract produced dose-related, significant analgesic effects against thermally induced nociceptive pain of the rat hind paw, in the fresh egg albumin-induced AI [17]. In our study, the ginger root capsule aqueous extract prolonged latency in the hot plate test (Table 6, Figure 5), so ginger might also be acting centrally. 

In the present study, the inflammation induction after carrageenan administration led to increased plasma levels of pro-oxidant parameters such as MDA, NOx, TOS and OSI and decreased plasmatic levels of the antioxidant parameters such as TAC and SH. Ginger root capsule extract administration provided a reduced antioxidant effect as the two doses slightly reduced the plasmatic levels of the above-mentioned pro-oxidant parameters and slightly improved the plasmatic levels of the evaluated antioxidant parameters. Diclofenac sodium administration was associated with a reduction of all the evaluated pro-oxidant parameters and elevation of all studied antioxidant parameters, more than GRCE alone. The association of GRCE with diclofenac sodium had an additional dose-dependent beneficial effect on all studied oxidative stress parameters (Table 5 and Table 6, Figure 4).

The release of neutrophil-derived free radicals is responsible for oxidative stress imbalance, which is specific to the second phase of edema induced by carrageenan [56]. Lipids are the biomolecules most involved in oxidative stress, as lipid peroxidation gives rise to several secondary products. Malondialdehyde is considered the principal and most studied product of polyunsaturated fatty acid peroxidation as it is regarded as a highly toxic molecule [57]. Diclofenac administration reduces serum lipid peroxidation [58], reduced the MDA plasma levels in a rat adjuvant arthritis model [59], and on carrageenan-induced paw edema [60]. Ginger has a similar effect as it inhibits lipid peroxidation and reduces MDA levels [61].

Nitric oxide (NO) is another major product of oxidative stress that plays a key role in the pathogenesis of inflammation. Under normal physiological conditions, it has beneficial effects in modulating vascular tone, as a vasodilator [62]. It can contribute to inflammatory damage if overproduced (by iNOS) together with excess superoxide anion, thus giving rise to harmful peroxynitrite (the so-called nitroxidative stress) [63]. Nitric oxide was observed to be involved in the pathogenesis of inflammatory disorders of the joints, gut and lungs. Therefore, NO inhibitors could represent an important therapeutic advance in managing inflammatory diseases, as different selective NO inhibitors might be helpful in treating NO-induced inflammation [64,65]. Diclofenac reduces inducible nitric oxide synthases (iNOS) expression in macrophages, decreasing NOx levels [66]. Inhibition of iNOS expression was suggested as a possible mechanism for NOx reduction after ginger powder supplementation [67].

Total oxidant status (TOS) is another pro-oxidant marker often used to estimate the overall oxidation state of the body [68], while TAC is an antioxidant marker used to evaluate the antioxidant capacity of the body [69]. It was already observed that diclofenac administration reduces TOS and increases TAC in carrageenan-induced paw edema inflammation in rats [70]. Ginger was observed to reduce TOS on renal ischemia/reperfusion injury in rat kidneys due to reduced oxidant substances excretion [71]. Ginger (*Zingiber officinale Roscoe*) administration was observed to increase TAC as a result of antioxidant defending capacity and decrease oxidative stress [72]. Moreover, ginger can be considered a storehouse of antioxidants as its bioactive ingredients such as gingerols, shogaols and zingerone were observed to have antioxidant activity by inhibiting oxidase enzymes such as xanthine oxidase [73]. 

A more precise biomarker reflecting oxidative stress is the OSI pro-oxidant marker, which can reflect an imbalance between antioxidants and pro-oxidation levels as it is defined as the ratio of the TOS level to the TAC level [74]. Ginger can influence the TAC and TOS ratio and therefore reduce the OSI index through the prevention of oxidation and nitration reactions induced by peroxynitrite, inhibition of xanthine oxidase responsible for the generation of reactive oxygen species, such as superoxide anion or inhibition of NO synthesis [75].

Thiols are a group of antioxidant molecules regarded as a useful defense system against biochemical alterations produced by oxidative stress [76]. Total thiol levels were increased after ginger administration as ginger was observed to possess high levels of biological thiols [77].

The low bioavailability and extensive phase II metabolism might be a limitation for the use of ginger in different pathologies and therefore new pharmaceutical forms for delivering ginger’s bioactive compounds are currently being developed [78]. For example, nanocarriers may further improve the beneficial effects of natural-based bioactive compounds as they protect the active compound from external injuries and internal pH variations [79,80].

### 4.3. Limitations of the Study and Call for Future Studies

No evaluations of the anti-inflammatory effects of GRCE associated with D were performed in this study since such measurements were out of our aim. Analyzation of the anti-inflammatory effects of GRCE in combination with diclofenac in acute inflammation is of real interest. Additional tests focused on the antinociceptive actions of ginger could be included in future studies. Moreover, the encapsulation of gingers’ active compounds in nanocarriers for targeted drug delivery represents a topic for future research.

## 5. Conclusions

Ginger root capsule extract in doses of 50 mg/kg b.w., 300 mg/kg b.w. or 2000 mg/kg b.w. were observed not to be toxic. Ginger root capsules associated with diclofenac might offer additional antioxidant and analgesic effects, in a dose-dependent manner in acute inflammation. The association of ginger root capsule extract with a low dose of diclofenac sodium might be a useful option to decrease diclofenac sodium doses used, as this combination seems to be helpful for oxidative stress and pain reduction, and mobility improvement in acute inflammation. Additional studies are needed to achieve clinical evaluation not only for the antioxidant but also for the anti-inflammatory and antinociceptive effects of ginger. 

## Figures and Tables

**Table 1:** Parameters of HPLC mobile phases elution gradient.

Time (min)	Flow (mL/min)	Mobile Phase A (%) ^1^	Mobile Phase B (%) ^2^
0	0.5	95	5
2	0.5	95–60	5–40
18	0.5	60–10	40–90
20	0.5	10	90
25	0.5	10–95	90–5
30	0.5	95	5

^1^ Mobile phase A: 0.1% acetic acid in distilled water, *v*/*v*; ^2^ mobile phase B: 0.1% acetic acid in acetonitrile, *v*/*v*.

**Table 2:** Description of applied test, measurements and outcomes.

Test	Method	Measurement and Interpretation [ref]
Motility	What? Observation for a period of 5 min	Score: 0 (rat walked with difficulty and avoided touching the toes of the inflamed paw to the floor); 1 (rat walked with little difficulty, but it touched the floor with the toe of the inflamed paw); or 2 (rat walked easily without any difficulties) [39]
Paw Pressure	What? Mechanical nociceptive response Device: an analgesy-meter (Ugo Basile, Milan, Italy) How: we applied a uniform increasing mechanical pressure on the rat’s right-hind paw (cut-off pressure = 500 g)	Latency response was recorded as the retraction of the rat’s paw [40]
Hot Plate	What? Heat sensitivity Device: Ugo Basile hot plate (Milan, Italy) heated to 55 °C How? Each rat was placed on the plate at 55 ± 0.1 °C	Time of latency was defined as the time between the moment when the animal was placed on the hot plate and the moment when the animal jumped off to avoid thermal pain or licked its hind paw. The cut-off was 20 sec set to prevent tissue damage. A decrease in paw withdrawal latency was interpreted as thermal hyperalgesia [41]

**Table 3:** Identification and quantification of main compounds in the ginger root ethanol extract by HPLC-DAD-ESI MS.

No	Rt (min)	UV Λmax (nm)	[M+H] + (*m*/*z*)	Tentative Identification	Concentration ^1^ μg/mL
1	23.93	220, 280	297	6-Gingerdiol	105.713
2	24.28	231, 280	295	6-Gingerol	443.182
3	24.78	229, 279	309	Methyl-6-gingerol	128.096
4	25.13	221, 281	323	8-Gingerol	360.897
5	25.38	226, 281	277	6-Shogaol	369.816
6	25.72	220, 254, 370	291	1-Dehydro-6-gingerdione	304.115
7	26.18	220, 281	351	10-Gingerol	525.418
8	26.56	229, 279	395	Methyl diacetoxy-6-gingerdiol	235.958
9	27.02	220, 253, 370	319	1-Dehydro-8-gingerdione	196.990
10	27.75	220, 278	409	Diacetoxy-8-gingerdiol	294.214
11	27.99	227, 281	393	Acetoxy-10-gingerol	140.897
12	28.33	220, 253, 370	347	1-Dehydro-10-gingerdione	219.324

^1^ Concentration is expressed as μg/mL gallic acid equivalent.

**Table 4:** Weight and biochemical evaluation for toxicity.

GRCE Dose	Rat	Weight (g)			ALT (U/L)	TB (mg/dL)	Creatinine (mg/dL)	Urea (mg/dL)
		Week 0	Week 1	Week 2				
50mg/kg b.w	1	302	310	322	65	0.7	0.44	39
	2	306	308	318	55	0.6	0.43	40
	3	310	320	326	54	0.4	0.47	38
300 mg/kg b.w	1	301	315	325	47	0.5	0.43	34
	2	303	310	320	48	0.6	0.38	40
	3	307	315	328	60	0.4	0.35	37
2000 mg/kg b.w	1	311	316	322	50	0.4	0.38	43
	2	307	317	328	60	0.6	0.36	37
	3	303	309	319	47	0.5	0.46	35
GRCE = ginger root capsules extract, ALT = alanine aminotransferase, TB = total bilirubin.								

**Table 5:** Serum levels of evaluated biomarkers, paw pressure and hot plate tests for the control (C), acute inflammation (AI) and acute inflammation treated with diclofenac (AI-D) groups.

Parameter	C Group	AI Group	AI-D Group	C vs. AI *	AI vs. AI-D *
**MDA (nmol/L)**	3.3 (0.5)	7.2 (0.4)	5.4 (0.4)	−18.3 (<0.0001)	9.1 (<0.0001)
**NOx (μmol/L)**	14.0 (3.4)	35.6 (6.6)	26 (2.6)	−8.2 (<0.0001)	3.8 (0.0019)
**TOS (μmol H_2_O_2_/eq/L)**	10.2 (3.4)	30.7 (8.9)	18 (5.3)	−6.0 (<0.0001)	3.5 (0.0039)
**TAC (mmol Trolox eq/L)**	1.2 (0.1)	0.8 (0.1)	0.9 (0.1)	8.0 (<0.0001)	−2.5 (0.0244)
**Total thiols (μmol/L)**	440.5 (33.3)	359.5 (23.5)	389.3 (37)	5.6 (0.0001)	−1.9 (0.0756)
**OSI**	8.9 (3.2)	40.4 (9.9)	20.6 (7.3)	−8.6 (<0.0001)	4.6 (0.0004)
**Paw pressure test**					
1 h	8.0 (0.4)	6.1 (0.3)	6.7 (0.7)	10 (<0.0001)	−2.3 (0.0367)
3 h	7.8 (0.5)	3.9 (0.2)	5.6 (0.3)	18.6 (<0.0001)	−11.8 (<0.0001)
5 h	7.8 (0.5)	2.7 (0.3)	4.2 (0.2)	26 (<0.0001)	−13 (<0.0001)
7 h	7.8 (0.7)	3.8 (0.3)	5.3 (0.5)	14.6 (<0.0001)	−6.9 (<0.0001)
24 h	7.7 (0.6)	5.1 (0.5)	6.2 (0.2)	9.7 (<0.0001)	−6.2 (<0.0001)
**Hot plate test**					
1 h	8.5 (0.6)	6.0 (0.6)	6.7 (0.7)	8.6 (<0.0001)	−2.1 (0.0513)
3 h	8.8 (0.4)	5.0 (0.6)	7.0 (0.4)	14.6 (<0.0001)	−7.5 (<0.0001)
5 h	9.1 (0.5)	3.1 (0.4)	6.7 (0.7)	25.1 (<0.0001)	−12.8 (<0.0001)
7 h	9.6 (0.8)	4.1 (0.5)	7.0 (0.3)	17.5 (<0.0001)	−15.8 (<0.0001)
24 h	9.4 (1.3)	5.3 (0.4)	7.1 (0.2)	8.4 (<0.0001)	−10.7 (<0.0001)

Data are expressed as mean (standard deviation); * *t*-test for independent samples: test statistics (*p*-value).

**Table 6:** Serum levels of evaluated biomarkers, paw pressure and hot plate tests for the control with GRCE and GRCE-D and comparison between all groups.

Parameter	AI-GRCE100	AI-GRCE200	AI-GRCE100-D	AI-GRCE200-D	Stat. (*p*-Value) *
**MDA (nmol/L)**	6.5 (0.6)	5.5 (0.4)	4.7 (0.4)	4.1 (0.4)	52.0 (<0.0001) ^a^
**NOx (μmol/L)**	30.6 (5)	24.4 (3.3)	20.8 (3.6)	16.5 (2.3)	21.4 (<0.0001) ^b^
**TOS (μmol H_2_O_2_/eq/L)**	23.9 (2.2)	21.5 (6.6)	14.2 (3.1)	12.2 (3.1)	12.6 (<0.0001) ^c^
**TAC (mmol Trolox eq/L)**	1.1 (0.1)	1.1 (0.1)	1.2 (0.1)	1.4 (0.1)	35.6 (<0.0001) ^d^
**Total thiols (μmol/L)**	395.8 (26.1)	418.3 (28.8)	479.3 (51.5)	522.8 (64.2)	17.8 (<0.0010) ^e^
**OSI**	22.7 (3.5)	19 (6.1)	11.5 (2.6)	8.5 (2.1)	28.6 (<0.0001) ^f^
**Paw pressure test**					
1 h	6 (0.9)	6.3 (0.5)	6.8 (0.3)	7.2 (0.6)	5.1 (0.0009) ^g^
3 h	4.3 (0.4)	4.7 (0.4)	6 (0.3)	6.3 (0.4)	64.8 (<0.0001) ^i^
5 h	3.3 (0.2)	3.7 (0.5)	4.7 (0.5)	5.3 (0.5)	50.8 (<0.0001) ^j^
7 h	4.2 (0.4)	5.1 (0.4)	5.5 (0.4)	6.1 (0.5)	33.0 (<0.0001) ^k^
24 h	5.4 (0.4)	5.8 (0.3)	6.7 (0.4)	6.7 (0.4)	25.1 (<0.0001) ^l^
**Hot plate test**					
1 h	6.1 (0.4)	6.1 (0.3)	7.5 (0.5)	7.6 (0.8)	12.1 (<0.0001) ^m^
3 h	5.3 (0.3)	5.6 (0.6)	7.7 (0.3)	8.4 (0.7)	57.3 (<0.0001) ^n^
5 h	3.9 (0.4)	4.9 (0.4)	7.5 (0.6)	8.6 (0.5)	137.3 (<0.0001) ^o^
7 h	5.1 (0.4)	5.5 (0.5)	7.6 (0.6)	8.6 (0.4)	123.6 (<0.0001) ^p^
24 h	5.8 (0.2)	6.0 (0.4)	7.6 (0.5)	8.9 (0.7)	70.0 (<0.0001) ^r^

Data are expressed as mean (standard deviation); * ANOVA test: F statistics (*p*-value). Groups: 1 = C, 2 = AI, 3 = AI-D, 4 = AI-GRCE100, 5 = AI-GRCE200, 6 = AI-GRCE100-D, 7 = AI-GRCE200-D; *p*-values of Scheffe test as post hoc ANOVA (adjusted significance level of 0.0083): (a) *p*-values < 0.003 for 2 vs. 3, 5, 6, 7 and 3 vs. 4, 7 and 4 vs. 5, 6, 7 and 5 vs. 7; (b) *p*-values < 0.004 for 2 vs. 3, 5, 6, 7 and 3 vs. 7 and 4 vs. 6, 7; (c) *p*-values < 0.007 for 2 vs. 3, 6, 7 and 4 vs. 7; (d) *p*-values < 0.0098 for 2 vs. 4, 5, 6, 7 and 3 vs. 5, 6, 7 and 4 vs. 7 anI5 vs. 7; (e) *p*-values < 0.007 2 vs. 6, 7 and 3 vs. 6, 7 and 4 vs. 7 and 5 vs. 7; (f) *p*-values < 0.002 for 2 vs. 3, 4, 5, 6, 7 and 4 vs. 7 and *p*-values < 0.05 for 3 vs. 7 and 5 vs. 7; (g) *p*-values < 0.03 for 2 vs. 7 and 4 vs. 7; (i) *p*-values < 0.007 for 2 vs. 3, 5, 6, 7 and 3 vs. 4, 5 and 4 vs. 6, 7 and 5 vs. 6, 7 and *p*-value < 0.03 for 3 vs. 7; (j) *p*-values < 0.0005 for 2 vs. 3, 5, 6, 7 and 3 vs. 4, 7 and 4 vs. 6, 7 and 5 vs. 6, 7; (k) *p*-values < 0.002 for 2 vs. 3, 5, 6, 7 and 3 vs. 4 and 4 vs. 6, 7 and 5 vs. 7 and *p*-value = 0.0090 for 4 vs. 5; (l) *p*-values < 0.004 for 2 vs. 3, 6, 7 and 4 vs. 6, 7 and 5 vs. 6, 7; (m) *p*-values < 0.003 for 2 vs. 6, 7 and 4 vs. 6, 7 and 5 vs. 6, 7; (n) *p*-values < 0.0005 for 2 vs. 3, 5, 6 and 3 vs. 4, 5, 7 and 4 vs. 6, 7 and 5 vs. 6, 7; (o) *p*-values < 0.0001 for 2 vs. 3, 5, 6, 7 and 3 vs. 4, 5, 7 and 4 vs. 6, 7 and 5 vs. 6, 7 and *p*-value = 0.0086 for 6 vs. 7 and *p*-value = 0.0315 for 4 vs. 5; (p) *p*-values < 0.0007 for 2 vs. 3, 4, 5, 6, 7 and 3 vs. 4, 5, 7 and 4 vs. 6, 7 and 5 vs. 6, 7 I 6 vs. 7; and (r) *p*-values < 0.0015 for 2 vs. 3, 6, 7 and 3 vs. 4, 5, 7 and 4 vs. 6, 7 and 5 vs. 6, 7 and 6 vs. 7.

## Data Availability

The experimental data will not be publicly available until the associated Ph.D. thesis is published. It can be obtained upon reasonable request addressed to Ioana Boarescu (e-mail: ioana.chirila.boarescu@elearn.umfcluj.ro).
